# SDF1 gradient associates with the distribution of c-Kit+ cardiac cells in the heart

**DOI:** 10.1038/s41598-018-19417-8

**Published:** 2018-01-18

**Authors:** Outi Renko, Anna-Maria Tolonen, Jaana Rysä, Johanna Magga, Erja Mustonen, Heikki Ruskoaho, Raisa Serpi

**Affiliations:** 1 Research Unit of Biomedicine, Department of Pharmacology and Toxicology, University of Oulu, Oulu, Finland; 2 School of Pharmacy, University of Eastern Finland, Kuopio, Finland; 3 Division of Pharmacology and Pharmacotherapy, University of Helsinki, Helsinki, Finland; 4 Biocenter Oulu, Faculty of Biochemistry and Molecular Medicine, Oulu Center for Cell-Matrix Research, University of Oulu, Oulu, Finland

## Abstract

Identification of the adult cardiac stem cells (CSCs) has offered new therapeutic possibilities for treating ischemic myocardium. CSCs positive for the cell surface antigen c-Kit are known as the primary source for cardiac regeneration. Accumulating evidence shows that chemokines play important roles in stem cell homing. Here we investigated molecular targets to be utilized in modulating the mobility of endogenous CSCs. In a four week follow-up after experimental acute myocardial infarction (AMI) with ligation of the left anterior descending (LAD) coronary artery of Sprague-Dawley rats c-Kit+ CSCs redistributed in the heart. The number of c-Kit+ CSCs in the atrial c-Kit niche was diminished, whereas increased amount was observed in the left ventricle and apex. This was associated with increased expression of stromal cell-derived factor 1 alpha (SDF1α), and a significant positive correlation was found between c-Kit+ CSCs and SDF1α expression in the heart. Moreover, the migratory capacity of isolated c-Kit+ CSCs was induced by SDF1 treatment *in vitro*. We conclude that upregulation of SDF1α after AMI associates with increased expression of endogenous c-Kit+ CSCs in the injury area, and show induced migration of c-Kit+ cells by SDF1.

## Introduction

Heart failure (HF) is a major health problem affecting more than 23 million people globally^1^ with a still increasing prevalence because of the aging of the population. The most common cause of HF is myocardial infarction (MI). Against the old dogma of the heart being incapable of renewal after injury or upon aging, recent studies have shown that it actually is capable of new cardiomyocyte formation with varying regenerative potential^2^. Data suggests that a pool of cardiomyocytes is cycling in the normal and pathological human heart^3–6^.

Identification of tissue-specific adult stem cells, cardiac stem cells (CSCs) from rats^7^, mice^8,9^, dogs^10^ and humans^11^ has led to the development of cell therapy strategies for enhancement of the growth response of the injured myocardium. CSCs positive for the cell surface antigen c-Kit have been reported as the primary source for cardiac regeneration after injury^7,12,13^, although their capacity to generate new cardiomyocytes is still not clear. There are a number of reports describing formation of adult cardiomyocytes from c-Kit+ CSCs *in vivo*^7,14,15^. However, several other papers report no significant differentiation of transplanted c-Kit+ CSCs into mature cardiomyocytes^16–18^ indicating that other factors, such as the paracrine mechanisms are responsible for the functional improvement. Also, the cardiomyogenic nature of endogenous c-Kit+ CSCs has been questioned^19,20^. The role and mechanism of action of c-Kit+ CSCs in cardiac regeneration thus remains controversial. One plausible explanation for the discrepant results is the expression of c-Kit receptor in different pools of cardiac progenitors, some of which are capable of cardiomyogenesis and others not^21^. In a healthy human heart the endogenous c-Kit+ CSCs are known to be present in the left and right ventricle of the heart but tend to accumulate in the atria, especially in the right atria^22^ of the heart, corresponding to the area exposed to lower levels of hemodynamic stress. The origin of the c-Kit+ CSCs, whether they are organ specific or derive from colonization of hematopoietic stem cells (HSCs) from the bone marrow to the myocardium, is also still an open question.

In heart, the CSCs are stored in niches that constitute the microenvironment in which they are maintained in quiescent state^12^ and after activation can replicate and migrate to injury sites to differentiate and acquire the adult phenotype. Evidence is accumulating that growth factors and chemokines play an important role in stem cell signaling and their homing to injured myocardium^23–25^. A chemokine (C-X-C motif) receptor 4 (CXCR4)^26–28^, and its ligand, stromal cell-derived factor 1 (SDF1) alpha, originally identified as a molecule secreted in bone marrow stromal cell lines attracting and stimulating the growth of B-cells^29–31^, provide attraction to many different cell types during development and adult life^32^, attracting for example lymphocytes and hematopoietic stem cells. The guidance of many different cell types to different targets in close proximity of each other by SDF1 during development suggests tight regulation of the spatial and temporal distribution of SDF1, however, how such a control is achieved is currently not well known^32^. In the heart, SDF1α has been shown to recruit CXCR4 expressing stem cells including HSCs and CSCs^33,34^, and hepatocyte growth factor (HGF)^35,36^, fibroblast growth factor-2 (FGF-2)^37^ and insulin growth factor-1 (IGF-1)^38^ have been shown to activate CSCs. These new data offer opportunities to find ways to manipulate the cells using chemokines to achieve better homing and regenerative capacity.

The aim of this study was to find molecular targets to be utilized in modulating the mobility of endogenous CSCs.

## Results

### Ligation of the LAD caused decreased function and remodeling of the left ventricle

**Figure 1:**
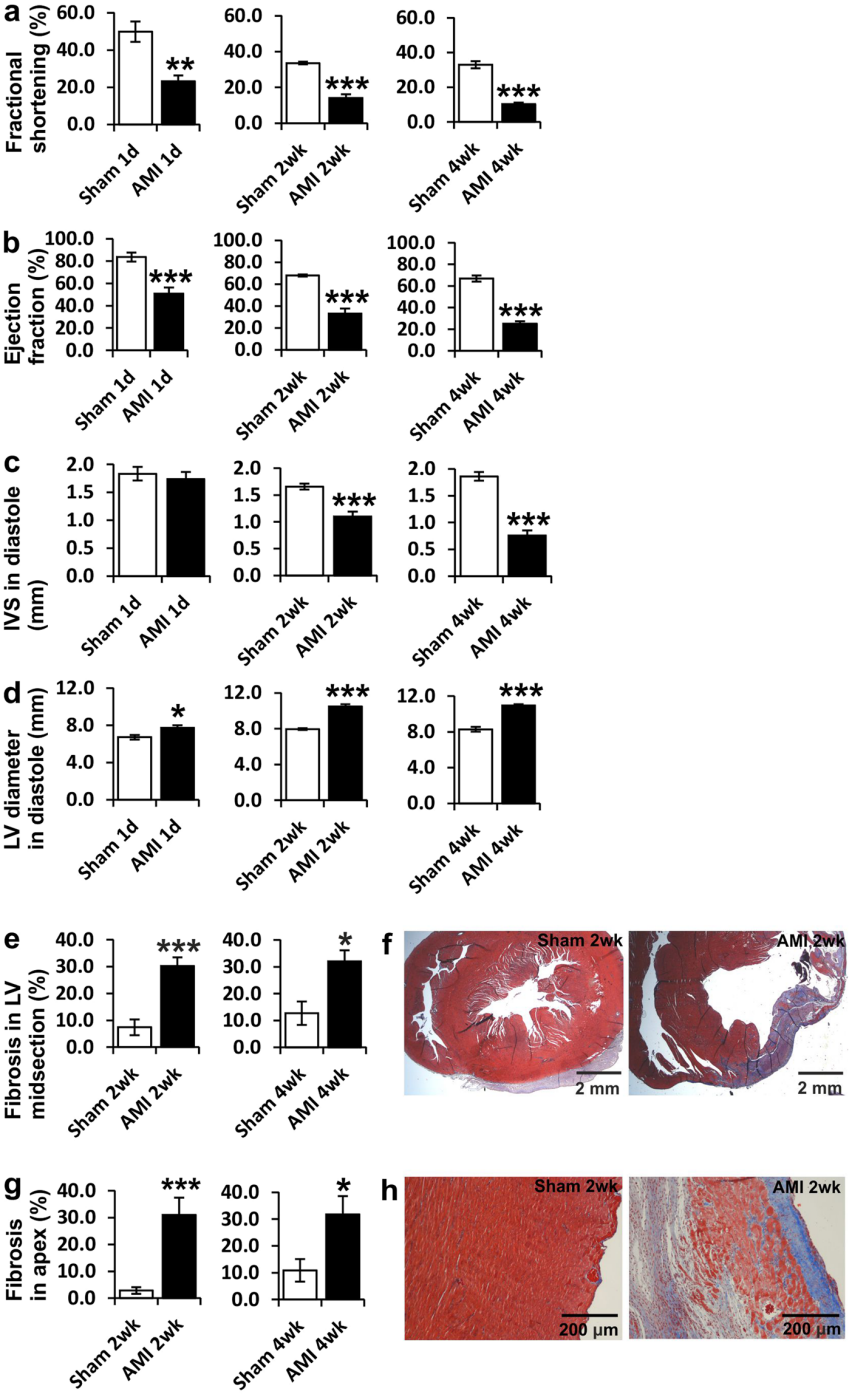
Effects of ligation of the LAD on LV structure and function. (**a**) LV fractional shortening (%) and (**b**) LV ejection fraction (%) 1 day, 2 weeks or 4 weeks after LAD-ligation compared to sham treated rats. (**c**) Thickness of interventricular septum (IVS) (mm) and (**d**) LV diameter (mm) in diastole 1 day, 2 weeks or 4 weeks after LAD-ligation compared to sham. (**e**) Percentage of fibrotic area in LV 2 or 4 weeks after LAD-ligation compared to sham and (**f**) representative pictures. (**g**) Percentage of fibrotic area in apex 2 or 4 weeks after LAD-ligation compared to sham and (**h**) representative pictures. N = 4–7 for all groups. Student’s *t* test was used for comparison between two groups. *P < 0.05, **P < 0.01, ***P < 0.001. Scale bars 2 mm (**f**) and 200 μm (**h**).

**Figure 2:**
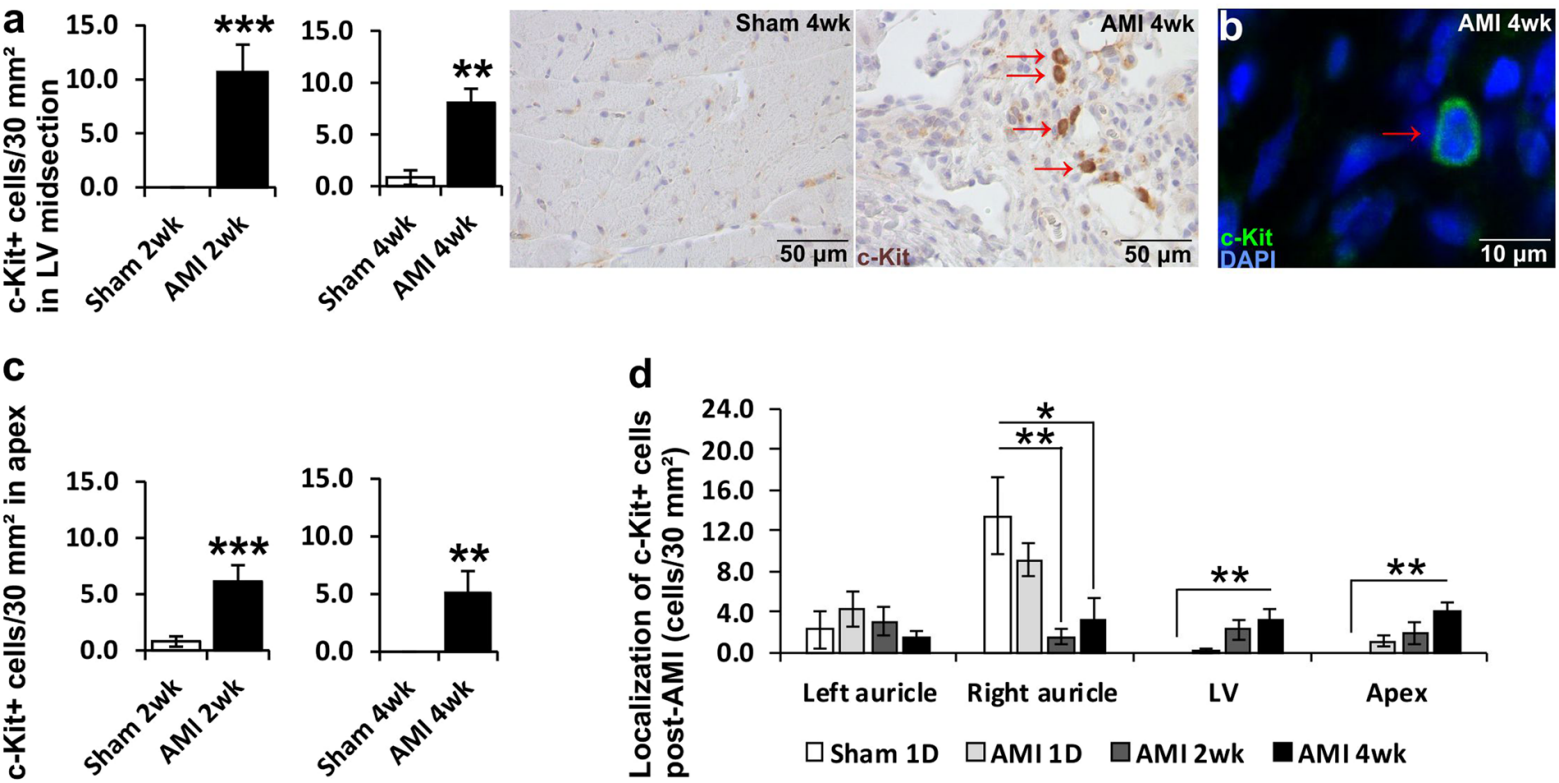
Localization of c-Kit+ cells in the heart. (**a**) Number of c-Kit+ cells in LV midsection and representative pictures of c-Kit+ cells in sham-treated LV and LV 4 weeks after AMI (scale bar 50 μm), (**b**) representative immunofluorescence picture of a c-Kit+ cell in LV from 4 week AMI sample (scale bar 10 μm). (**c**) Number of c-Kit+ cells in apex 2 or 4 weeks after LAD-ligation compared to sham treated rats. (**d**) Number of c-Kit+ cells in left and right auricle, LV midsection and apex of the heart in sham treated rats after 1 day or 1 day, 2 weeks or 4 weeks after LAD-ligation. N = 5–7 for all groups. Mann–Whitney *U* test was used for comparison between two groups and Kruskal–Wallis one-way analysis of variance for comparison with multiple groups. *P < 0.05, **P < 0.01, ***P < 0.001.

Characteristics representative for acute myocardial infarction (AMI) were observed after the ligation of the left anterior descending (LAD) coronary artery of 2-month-old young adult Sprague-Dawley rats. Left ventricular fractional shortening (FS) and ejection fraction (EF) decreased significantly at all time points (1 day, 2 weeks and 4 weeks) compared to sham-treated animals (Fig. 1a,b). The decrease in contractility of the heart was associated with myocardial remodeling, as reflected by significant thinning of the interventricular septum (IVS) in diastole and increase in the left ventricular (LV) diameter (Fig. 1c,d and f). Also, accumulation of connective tissue was greatly increased in the midsection of the left ventricle at 2 and 4 weeks after AMI (Fig. 1e,f) and in the apex of the heart at 2 and 4 weeks after the ligation (Fig. 1g,h). The number of Tunel+ apoptotic cells was significantly increased in the midsection of the left ventricle of the heart 2 weeks post-ligation and a tendency of increased number remained at 4 weeks (Supplementary Fig. 1). There was also a significant increase in the number of Tunel+ cell in the apex of the heart at 2 and 4 weeks after AMI (Supplementary Fig. 1). Number of peripheral blood white blood cells (WBC) was increased 2 weeks post-AMI (Supplementary Fig. 1). Also, significant hypertrophy of the cardiomyocytes (Supplementary Fig. 1) as well as nuclear hypertrophy (seen in sections in Fig. 2a) were observed in LV midsection and in apex of the heart.

### Altered localization of c-Kit+ cardiac stem cells in the heart after AMI *in vivo*

Histological analysis revealed significantly increased number of c-Kit+ CSCs in the infarct border zone in the midsection of LV (P < 0.001 at 2 weeks and P < 0.01 at 4 weeks) as well as in the apex of left ventricle 2 (P < 0.001) and 4 weeks (P < 0.01) after the ligation of the LAD (Fig. 2a–c). Increased expression of c-Kit protein was seen also with Western blotting (P < 0.05) (Supplementary Fig. 2). A more detailed analysis of the localization of c-Kit+ CSCs in the heart before and 1 day and 2 and 4 weeks after the AMI showed that the c-Kit+ CSCs resided mostly on the right auricle of the heart at the baseline, and after the AMI their number in the right auricle diminished, whereas it was significantly increased in the midsection of the left ventricle and the apex of the heart (Fig. 2d). Even though the number of c-Kit+ CSCs in the auricles decreased after MI, their number still remained at the same level with the increased number of CSCs in the LV and apex after MI. To verify that the c-Kit+ cells were not mast cells, a double staining with toluidine blue was performed, showing no co-localization of the two stains on same cells (Supplementary Fig. 1).

### Increased expression of SDF1α in the heart post-AMI

**Figure 3:**
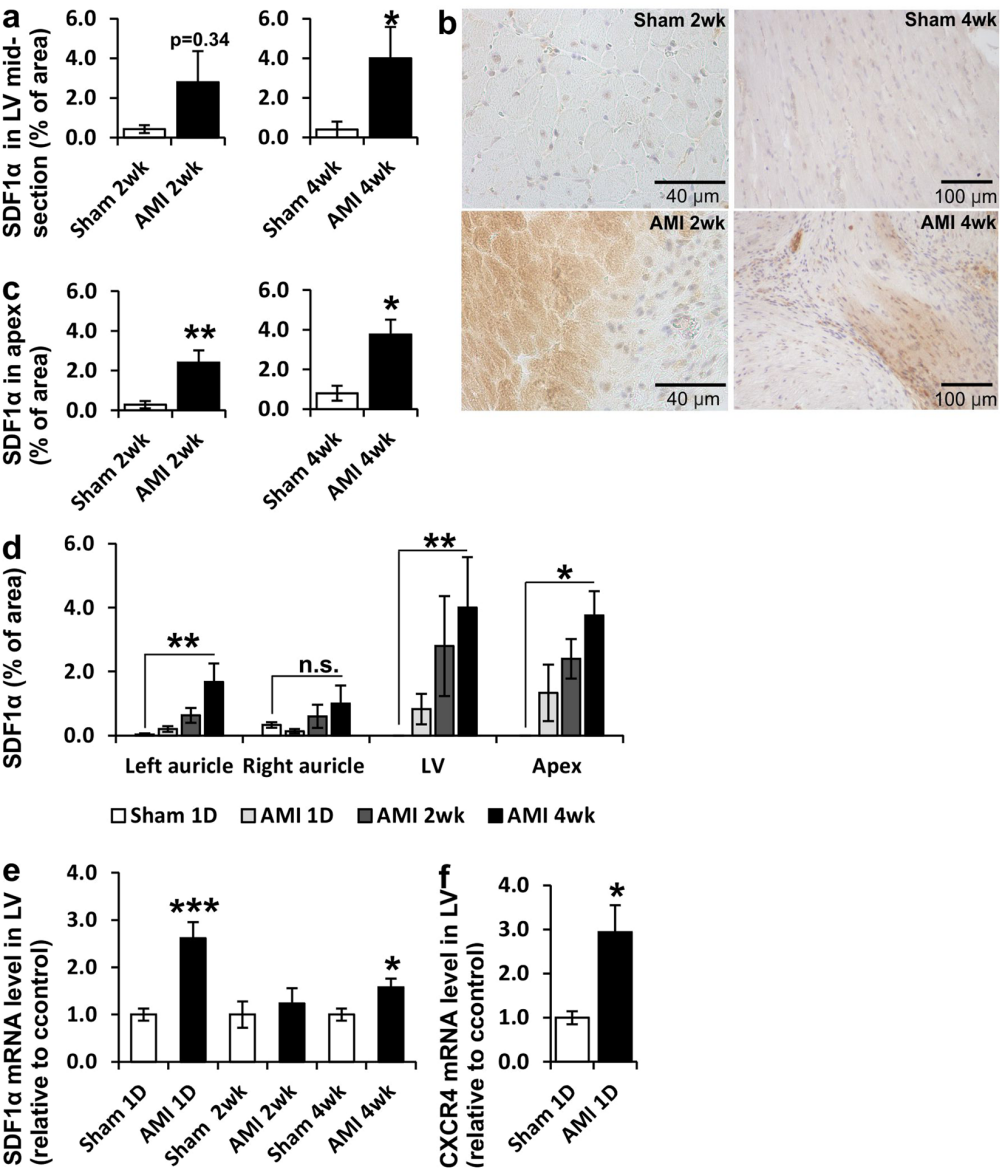
Expression of SDF1α in the heart. (**a,b**) SDF1α protein in infarct region of LV midsection and (**c**) apex 2 or 4 weeks after LAD-ligation compared to sham treated rats. (**d**) Expression of SDF1α protein in left and right auricle, LV midsection and apex of the heart in sham treated rats after 1 day or 1 day, 2 weeks or 4 weeks after LAD-ligation. (**e**) SDF1α mRNA in LV relative to control 1 day, 2 weeks or 4 weeks after LAD-ligation. (**f**) CXCR4 mRNA in LV relative to control 1 day after LAD-ligation. N = 5–7 for all groups. Mann–Whitney *U* test was used for comparison between two groups and Kruskal–Wallis one-way analysis of variance for comparison with multiple groups. *P < 0.05, **P < 0.01, ***P < 0.001. Scale bars 40 and 100 μm.

Expression of SDF1α was increased in the hearts after ligation of the LAD compared to the hearts of the sham-treated animals. In the midsection of the left ventricle, a significant increase in the expression was detected at 4 weeks after the ligation of the LAD (Fig. 3a,b), and in the apex of the heart the increase was statistically significant at 2 and at 4 weeks after the ligation (Fig. 3c). A small non-significant upregulation was also seen with Western blotting analysis from the 2 week time-point (Supplementary Fig. 2). From the 1 day and 4 week time-points no SDF1α protein was detected by immunoblotting, and the protein-level upregulation of SDF1α therefore remains a debatable issue. When different compartments of the heart were analyzed, a slightly induced expression of SDF1α was observed 4 weeks after the ligation in the auricles whereas in the left ventricle as well as in the apex of the heart the expression of SDF1α was more robustly increased (Fig. 3d). Variation in the expression level between animals was large, as can be seen in the large error bars. SDF1α expression was increased also on mRNA level; a 2.6-fold increase was seen 1 day after AMI (P < 0.001) and still at 4 weeks after the ligation a significant increase was seen (P < 0.05) (Fig. 3e). Also the expression of CXCR4 was upregulated 3-fold (P < 0.05) at the 1 day time point (Fig. 3f).

**Figure 4:**
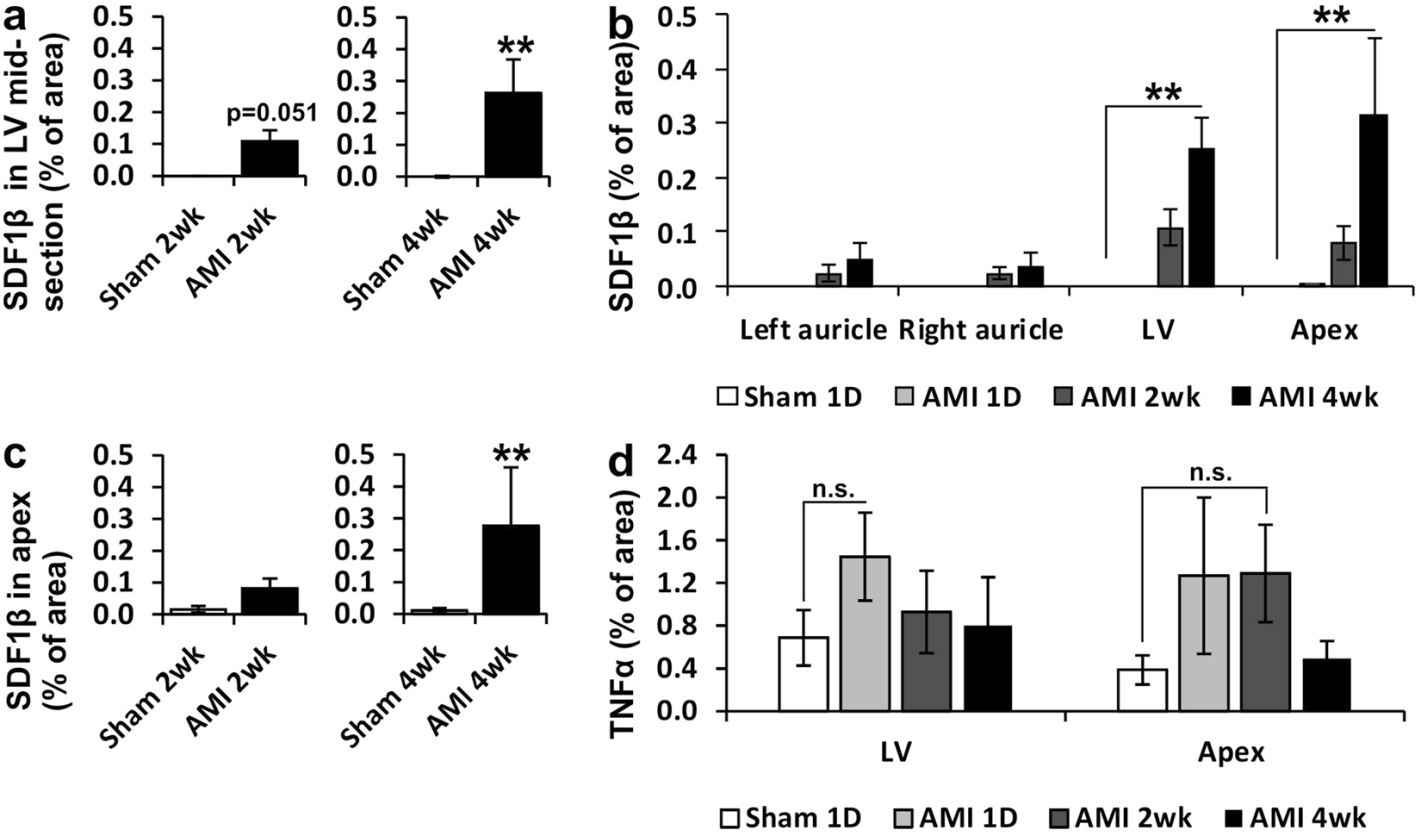
Expression of SDF1β and TNFα in the heart. (**a**) SDF1β in LV midsection and (**b**) expression of SDF1β in left and right auricle, LV midsection and apex of the heart in rats 1 day, 2 weeks or 4 weeks after the ligation of LAD compared to sham treated rats. (**c**) SDF1β expression in apex of the heart 2 or 4 weeks after LAD-ligation compared to sham treated rats and (**d**) expression of TNFα in LV midsection and apex 1 day, 2 weeks or 4 weeks after LAD-ligation. N = 5–7 for all groups. Mann–Whitney *U* test was used for comparison between two groups and Kruskal–Wallis one-way analysis of variance for comparison with multiple groups. *P < 0.05, **P < 0.01.

Of the other studied cytokines putatively able to affect the homing of CSCs, also the expression of SDF1β was increased in a similar manner after the ligation of the LAD, although the expression level was lower compared to the SDF1α (Fig. 4a–c). The expression of tumor necrosis factor α (TNFα) was slightly but not significantly increased at day 1 and 2 weeks after the AMI, but no difference was observed at 4-weeks (Fig. 4d).

### Increased migration of c-Kit+ CSCs by SDF1α *in vitro* and positive correlation between the number of c-Kit+ CSCs and SDF1α expression *in vivo*

**Figure 5:**
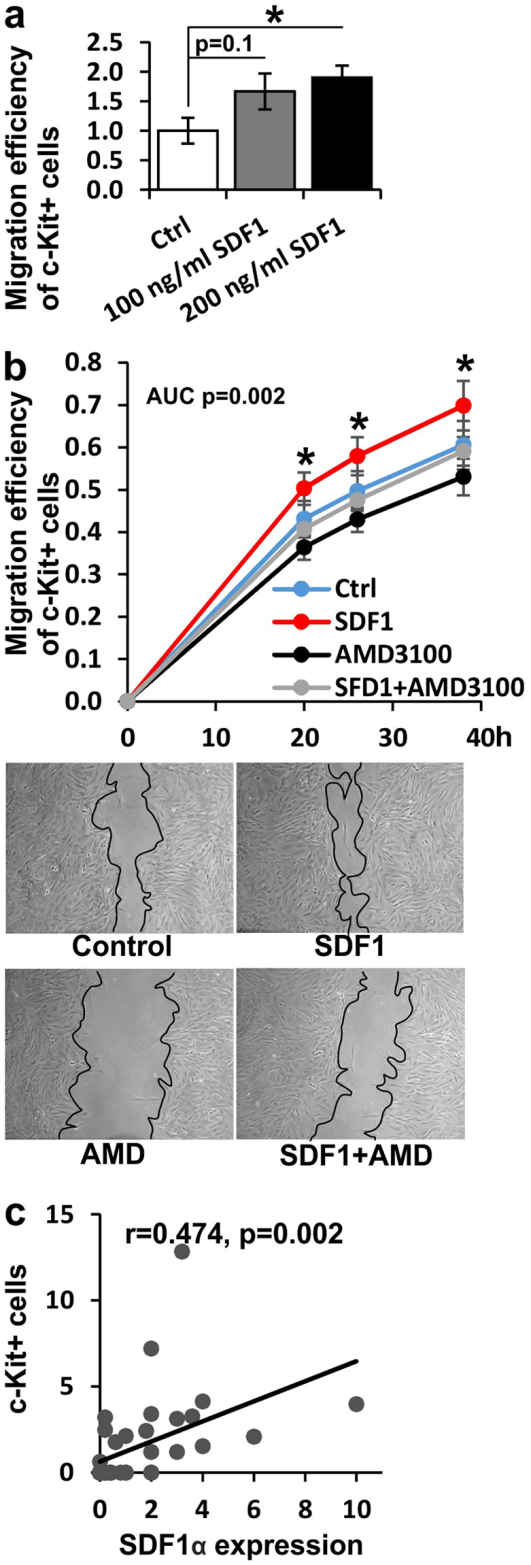
Effect of SDF1 on the migration of c-Kit+ cells. (**a**) Migration of c-Kit+ cells isolated from the MI border zone treated with 100 or 200 ng/ml SDF1 or vehicle control (N = 6 for all groups) and (**b**) with SDF1 and/or small-molecule inhibitor of CXCR4 AMD3100 or vehicle control (N = 9 for all groups). (**c**) Correlation between SDF1α expression and number of c-Kit+ cells. Student’s *t* test was used for comparison between two groups and areas under the curve (AUC) were calculated by the summary measures method. *P < 0.05.

Migration of isolated c-Kit+ CSCs was increased by SDF1α treatment *in vitro*. When CSCs isolated from the infarcted border zone in the mid-region of LV were treated with 100 or 200 ng/ml SDF1α in a wound-healing assay, cell migration was significantly increased in a dose-dependent manner (Fig. 5a). Likewise, the migration of cells isolated from the apex was increased (0.8 ± 0.2 with 100 ng/ml SDF1α and 1.5 ± 0.1 with 200 ng/ml SDF1α, P < 0.05). Importantly, treatment of the CSCs in the wound-healing assay with a small-molecule inhibitor of CXCR4 (AMD3100) was able to abolish the increased migration of CSCs induced by SDF1 treatment (SDF1 vs. AMD3100 at 20, 26 and 38 hours in culture, P < 0.05; the difference in the area under the curve between SDF1 and AMD3100 treated CSCs, P < 0.01) (Fig. 5b). A positive correlation was found between the number of c-Kit+ CSCs and the expression of SDF1α protein in the heart *in vivo* (R = 0.474, P < 0.01, Fig. 5c).

## Discussion

SDF1 is known to mediate the trafficking and homing of stem cells to bone marrow^39,40^ by binding to CXCR4 on circulating cells^41,42^. *In vitro*, cardiomyocytes transfected with SDF1α significantly increase the SDF1α concentration in culture media, and subsequently attract more CSC migration^33^. In *in vivo* mouse infarction model, the overexpression of SDF1α in the infarcted area results in more CSC retention to the infarcted myocardium^33^. Transplantation of syngeneic cardiac fibroblasts transfected to express SDF1 into myocardium has also been shown to induce homing of CD117/c-Kit+ hematopoietic progenitor cells to injured myocardium^34^. These data indicate that overexpression of SDF1α is able to enhance CSC migration and engraftment to the heart.

Hypoxia-inducible factor 1 (HIF-1) is a transcription factor that is expressed in response to a decrease in the partial pressure of cellular oxygen and activates genes involved in angiogenesis, glycolysis, and erythropoiesis^43,44^. In a model of soft-tissue ischemia in athymic nude mice it was shown that SDF1 expression is regulated by HIF-1 in endothelial cells, resulting in expression of SDF1 in ischemic tissue in direct proportion to reduced oxygen tension^45^. The HIF-1-mediated SDF1 expression increased the adhesion, migration and homing of circulating CXCR4+ progenitor cells to ischemic tissue^45^. Also, treatment of mice with dimethyloxalylglycine (DMOG), a prolyl-4-hydroxylase inhibitor, which stabilizes HIF-1α, was shown to increase the expression of cardiomyocyte CXCR4 associated with significant increase in cardiac progenitor cell recruitment to the heart 7 days after MI^46^. The recruitment-response was decreased in cardiomyocyte-CXCR4-null mice^46^. These data would suggest that induction of SDF1 expression via HIF-1 can directly guide regenerative progenitor cells to the injured area. HIF-1 is upregulated in the peri-infarct area of left ventricle after short-term ischemia in rats^47^ and after myocardial infarction in mice^48^ and humans^49^. The expression of HIF-1 protein is seen throughout areas of infarcted or ischemic myocardium, especially in the nuclei of cardiomyocytes and endothelial cells lining small vessels, whereas no HIF-1 expression is seen in non-infarcted or non-ischemic myocardium^49^. We have shown earlier that activation of hypoxia response via inhibition of the main regulator of the stability of HIFα subunit, hypoxia-inducible factor prolyl 4-hydroxylase-2 (HIF-P4H-2), contributes to ischemic cardioprotection after myocardial infarction^50^. Apparently this protection is especially due to the upregulation of hypoxia response in the endothelial cells because the protective effect was reversed by blockage of Tie-2 signaling^50,51^.

Pressure and volume overload in heart *in vivo* is characterized by myocyte stretch leading to induction of the expression and secretion of cardiac atrial natriuretic peptides (atrial and B-type natriuretic peptides, ANP and BNP, respectively)^52^. Both exert potent diuretic, natriuretic, vasorelaxant, aldosterone-inhibiting, antifibrotic, and antihypertrophic effects that are mediated through their common receptor, guanylyl cyclase (GC)-A via the generation of intracellular cGMP and thereafter activation of protein kinase G (PKG). Both ANP and BNP via the activation of the GC-A/cGMP/PKG –signaling pathway have been shown to lead to increased differentiation of cardiac progenitor cells^53,54^. Also, blockade of PKG1-signaling was shown to strongly slow down cardiogenesis of mouse embryonic stem cells^55^ whereas overexpression of PKG1α on rat bone marrow derived mesenchymal stem cells (MSCs) was associated with higher release of multiple factors such as SDF1 and increased myogenic differentiation of the PKG1α-MSCs^56^. Accordingly, in a study with patients with persistent atrial fibrillation, increased ANP levels in the peripheral blood were shown to correlate with increase in the expression of SDF1α and CD34+ hematopoietic progenitor cells^57^. Importantly, hematopoietic progenitor cells from these patients had a greater tendency to differentiate into cells expressing cardiomyocyte markers^57^. Also, a role for the oxytocin-ANP system in promoting cardiomyocyte differentiation was suggested in a study where mouse P19 embryonic stem cells induced with oxytocin exhibited increased levels of ANP mRNA as well as cardiomyocyte markers^58^. Collectively, these data strongly suggest a role for natriuretic peptides in stem cell differentiation towards cardiac lineage, which is also supported by our finding of endogenous c-Kit expressing CSCs mainly in the atria and the decrease in their number after pressure overload caused by the experimental myocardial infarction.

**Figure 6:**
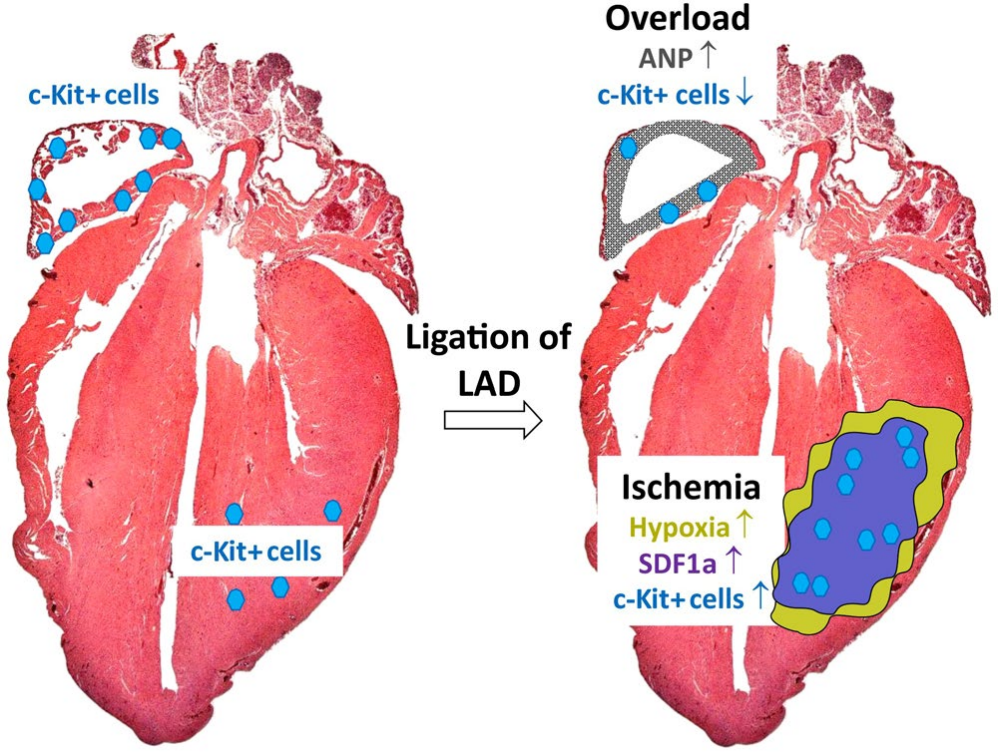
Hypothetical model of the effects of ligation of the LAD on localization of c-Kit+ CSCs in the heart. ANP; atrial natriuretic peptide^52^, SDF1α; stromal cell-derived factor 1 alpha^33,34^.

We propose here a hypothetical model in which the reduced oxygen tension in myocardium after myocardial infarction could be associated with the increased expression of SDF1 in the left ventricle and increased expression of c-Kit+ cells in the injury area (Fig. 6). The c-Kit expression might be regulated by the SDF1 gradient that is higher in the area closest to the injury site. The similar expression pattern of SDF1 expression (Fig. 3d) and c-Kit+ cells (Fig. 2d) in LV and apex of the heart after myocardial infarction in our study and the positive correlation between these parameters (Fig. 5c) supports this hypothesis. In the atrial tissue, on the other hand, the more pronounced challenge is not hypoxia but the volume overload, which via increased expression of natriuretic peptides and PKG-signaling may induce differentiation of cardiac c-Kit+ cells towards a more mature phenotype and hence diminish the c-Kit expression. Although the increased migration of c-Kit+ cells by SDF1 indicates a link between these parameters, our study has limitations. Gain- or loss-of-function studies, e.g. overexpressing or inhibiting SDF1 and their effect on the expression of c-Kit on CSCs and their migratory capacity would be needed to confirm the connection between upregulation of SDF1 expression and number of c-Kit+ cells. These aspects need to be addressed in the future studies. Also, it has to be kept in mind that culturing the isolated c-Kit+ cells *in vitro* may affect their phenotype and it is possible that they do not accurately represent the endogenous c-Kit+ CSCs.

In recent genetic lineage tracing studies with mouse heart it has been proposed that the cardiac c-Kit+ cells do not contribute particularly to cardiomyocyte formation in healthy or ischemic heart but are rather endothelial in nature^19,20,59,60^. On the other hand, c-Kit was shown to delineate multipotent cardiac progenitor cells of neural crest origin, fully able to contribute to new cardiomyocyte formation. These cells are distinct from the mesodermal c-Kit+ vasculogenic lineage demonstrating distinct cardiomyogenic and vasculogenic c-Kit+ lineages in the heart^61,62^. The relatively small contribution of the neural crest origin c-Kit+ cells to myocardium, was suggested to be related to a non-permissive cardiac milieu as opposed to the cells’ poor cardiomyogenic capacity. To summarize, whether via increased number of functional cardiomyocytes, overall support to the tissue by paracrine factors or by improved perfusion, the c-Kit+ cardiac cells appear to contribute beneficial effects to the heart. We have shown earlier in a rat pressure overload model that vascular endothelial growth factor-B (VEGF-B) gene transfer prevented the development of pressure overload induced diastolic dysfunction via increased c-Kit expression and proliferation as well as dilatation of pre-existing capillaries^63^. Also, in the mouse model with normoxic HIF-1 stabilization due to down regulation of HIF-P4H-2 the activation of hypoxia response preferentially in endothelial cells contributed to ischemic cardioprotection again via enlarged capillaries^50^. The role of c-Kit+ cardiac cells in endothelial differentiation in loaded myocardium is likely to possess options for myocardial recovery and will therefore warrant more studies. Finally, overexpressing SDF1 might serve as a means for attracting cells with regenerative capacity and hence improving heart failure symptoms in patients with ischemic cardiomyopathy^64^ and also deserves more attention in the future.

## Methods

### Myocardial infarction in rats

Male Sprague-Dawley rats weighing 250–300 g were used for the study following the 3 R rules of animal experimentation. Animal experiments were conducted according to the national regulations of the usage and welfare of laboratory animals and approved by the Animal Experiment Committee in the State Provincial Office of Southern Finland. Acute myocardial infarction (AMI) was produced by ligation of the left anterior descending coronary artery (LAD) as previously described^65^. Rats were anesthetized with 0.25 mg/kg medetomidine (Domitor vet) and 50 mg/kg ketamine (Ketaminol vet), and connected to the respirator through a tracheotomy. A left thoracotomy and pericardial incision was performed and the LAD was ligated. The sham-operated rats underwent the same surgical procedure without the ligation of LAD. After surgery, rats were treated with atipamezole (Antisedan vet) to reverse sedation. Carprofen (5 mg/kg, Rimadyl vet) and buprenorphine (0.05–0.2 mg/kg, Vetergesic vet) were administered as perioperative analgesia and rats were hydrated with 5 mL NaCl solution subcutaneously. Postoperative analgesia (carprofen once per day, buprenorphine twice per day) was administered for 3 days. Rats were sacrificed 1 day, 2 weeks or 4 weeks post-infarction.

### Echocardiography

Transthoracic echocardiography was performed using the Acuson Ultrasound System (SequoiaTM 512) and a 15-MHz linear transducer (15L8) (Acuson, MountainView, California, USA) as previously described^65^. Before examination, rats were sedated with 50 mg/kg ketamine and 10 mg/kg xylazine i.p. The rats were placed in the supine position and the normal body temperature was maintained during the examination by a warming pad and lamp. Using two-dimensional imaging, a short axis view of the left ventricle at the level of the papillary muscles was obtained, and a two dimensionally guided M-mode recording through the anterior and posterior walls of the LV was obtained. LV end-systolic and end-diastolic dimensions as well as the thickness of the interventricular septum (IVS) and posterior wall were measured from the M-mode tracings. LV FS and EF were calculated from the M-mode LV dimensions using the following equations: FS (%) = {(LVEDD-LVESD)/LVEDD} × 100, EF (%) = {(LVEDD)^3^– (LVESD)^3^/LVEDD^3^} × 100. For evaluation of LV diastolic function, mitral flow was recorded from an apical four-chamber view. Measurements of peak flow velocity of the early rapid diastolic filling wave (E) and late diastolic filling wave (A) were made and the E/A ratio was determined. The LV IVRT was also measured. All the measurements were made from three subsequent cycles and calculated as an average of these three measurements.

### Histology and image analysis

After echocardiography, animals were sacrificed and hearts collected. Hearts were fixed in phosphate-buffered 10% formalin (pH 7.0) and embedded in paraffin as described earlier^63^. Tissue sections were prepared from LV, apex and left and right auricles. For evaluation of the amount of interstitial fibrosis in myocardium and cardiomyocyte size, 5 µm thick transversal sections were cut at the level of papillary muscles and stained with Masson’s trichrome. Percentage of fibrotic area was quantified with a Nikon NIS-Elements BR 2.30 software from five representative fields of each heart cut on the LV midsection or from the apex of the heart. Primary antibody for c-Kit (sc-168, Santa Cruz Biotechnology or PA5-16770, ThermoFisher Scientific) was used to stain cardiac stem cells. The peroxidase label was developed by a peroxidase conjugated EnVision Detection Kit system (Dako) and the samples were counterstained with haematoxylin. To evaluate the number of c-Kit+ cells two separate sections of the whole tissue block (LV anterior wall, apex and left and right auricles) was scanned through and the number of c-Kit+ cells was counted. The area of counted sections was determined (Nikon NIS-Elements BR 2.30 software) and a number of c-Kit+ cells was related to the area (cells/30 mm^2^).

A CY2-conjugated secondary antibody (611-111-122, Rockland) was used for immunofluorescence microscopy to visualize the c-Kit+ cells in myocardium. SDF1α (5388, BioVision), SDF1β (ab25118, Abcam) and TNFα (HP8001, HyCult biotechnology) were used to detect the cytokine expression in myocardium. All histological analysis was done by investigators blinded to animal allocation.

### Real-time quantitative polymerase chain reaction

RNA was isolated from LV tissue with Trizol (Invitrogen). The cDNA was synthesized from 0.5 µg of total RNA (Transcriptor First-Strand cDNA Synthesis Kit, Roche). SDF1 and CXCR4 levels were measured by real-time quantitative (qPCR) analysis using TaqMan or SYBR chemistry on an ABI Prism 7700 Sequence Detection System (Applied Biosystems), and normalized to 18 S housekeeping gene. Oligonucleotide primer sequences used for mRNA quantitation by qPCR are following: SDF1 fluorogenic probe (FAM-TAMRA) 5′- CTGAGCTACAGATGCCCCTGCCGAT -3′; for 5′- ATCAGTGACGGTAAGCCAGTCA -3′; rev 5′- TGGCGACATGGCTCTCAAA -3′, CXCR4 for 5′- GACTGGCATAGTCGGCAATG -3′; rev 5′- AGAAGGGGAGTGTGATGACAAA -3′.

### Cell culture

C-Kit+ cardiac stem cells (CSCs) were isolated from infarction border zone in the midsection of LV or from apex 2 weeks after AMI as described^66^. Myocardial tissue was cut into pieces and enzyme-digested three times 5 min with 0.2% trypsin and 0.1% collagenase IV. Tissue explants were rinsed and cultivated in Iscove’s Modified Dulbecco’s Medium (IMDM), 10% fetal bovine serum, 2 mM L-glutamine, penicillin-streptomycin and 0.1 mM β-mercaptoethanol. Within 2–3 weeks, a layer of fibroblast-like cells was generated from adherent explants over which small, phase-bright cells migrated which were collected by pooling 2 min wash with PBS, 2 min wash with 0.53 mM EDTA, and 2 min wash with 0.05% trypsin and 0.53 mM EDTA at room temperature under visual control. Collected cells were seeded on poly-D-lysine-coated plates in 35% IMDM/65% Dulbecco’s Modified Eagle Medium (DMEM)-Ham F-12, 3.5% fetal bovine serum (FBS), 2% B27, 0.1 mM β-mercaptoethanol, 10 ng/ml epidermal growth factor (EGF), 20 ng/ml basic fibroblast growth factor (bFGF), 10 ng/ml cardiotrophin-1, 0.5 IU/ml thrombin, 2 mM L-glutamine and penicillin-streptomycin. Cells formed cardiospheres which were collected after 7 days and plated onto fibronectin-coated plates in IMDM, 10% fetal bovine serum, 2 mM L-glutamine, penicillin-streptomycin and 0.1 mM β-mercaptoethanol. After formation of monolayer culture, cells were trypsinized and used into migration assay.

### Migration assays

CSCs were plated onto fibronectin-coated 4-well plates and cultivated until a confluent monolayer culture. CSC migration was studied as wound healing assay as described earlier^67^. Wounds were created with a 1 ml pipet tip and the wound closure was monitored in the presence and absence of 100 or 200 ng/ml SDF1α. The wound healing rate, indicating cell migration rate, was determined as a comparison of initial wound area to the wound area after 16 h of cultivation. To study the effect of CXCR4 antagonist on CSC migration, cells were incubated immediately after wound creation in the presence of 200 ng/ml SDF1α (Peprotech), 200 nM AMD3100 (Sigma) or 200 ng/ml SDF1α and 200 nM AMD3100. Cells were pictured from the same site of the well at 0 h, 20 h, 26 h and 38 h after incubation. Wound healing rate was determined at each time point similarly as above.

### Statistical analysis

All data are shown as mean ± SEM. The data were analyzed with SPSS software using Student’s *t* test, Mann–Whitney *U* test or Kruskal–Wallis one-way analysis of variance with Bonferroni correction, when appropriate. Areas under the curve were calculated by the summary measures method. Pearson’s correlation coefficient was calculated to compare linear dependences between two variables. A P value of <0.05 was considered statistically significant.

## Electronic supplementary material

Supplementary information
