# Supplementary material for: SDF1 gradient associates with the distribution of c-Kit+ cardiac cells in the heart
Source: Sci Rep. 2018 Jan 18;8:1160. doi: 10.1038/s41598-018-19417-8 (PMC5773575; doi:10.1038/s41598-018-19417-8)

Supplementary Information **SREP-17-15225D**

SDF1 gradient associates with the distribution of c-Kit+ cardiac cells in the heart

Outi Renko[1], Anna-Maria Tolonen[1], Jaana Rysä[2], Johanna Magga[1], Erja Mustonen[1], Heikki Ruskoaho[3] and Raisa Serpi[4*]

[1]Institute of Biomedicine, Department of Pharmacology and Toxicology, University of Oulu, Oulu, Finland, [2]School of Pharmacy, University of Eastern Finland, [3]Division of Pharmacology and Pharmacotherapy, University of Helsinki, Helsinki, Finland and [4]Biocenter Oulu, Faculty of Biochemistry and Molecular Medicine, Oulu Center for Cell-Matrix Research, University of Oulu, Oulu, Finland

*Correspondence to: Raisa Serpi, PhD, Faculty of Biochemistry and Molecular Medicine, Aapistie 7, P.O. Box 5400, University of Oulu, FIN-90014, Finland. Tel. +358-(0)294-485833, Fax +358-8-5315037, Email raisa.serpi@oulu.fi

Supplementary Methods

Hearts were fixed in phosphate-buffered 10% formalin (pH 7.0) and embedded in paraffin and 5 µm thick transversal sections were prepared from LV midsection and apex of the heart. The ApopTag in situ apoptosis detection kit (S7100, Millipore) for 3´-end labeling of apoptotic DNA by terminal deoxynucleotidyl transferase –mediated dUTP nick end labeling (TUNEL) method was used to analyze the number of apoptotic cells. The cross-sectional area of cardiomyocytes was calculated as an average of 50 cardiomyocytes/section obtained from five representative fields of each Masson’s trichrome –stained section from epicardial and endocardial sides of the LV. Toluidine blue staining for mast cells was performed in combination of immunohistochemistry for c-Kit.

Total and nuclear proteins were extracted for Western blot analyses. Protein levels were detected using fluorescence with the Odyssey Fc imaging system (LI-COR Bioscience). The bands were quantified with ImageJ software. Primary antibodies for c-Kit (PA5-16770, ThermoFisher Scientific) and SDF1 (ab25118, Abcam) were used.

Supplementary Figure legends

Supplementary Figure 1. Apoptosis and cardiomyocyte hypertrophy on LV and apex of the heart after LAD-ligation. (**a-c**) Number of apoptotic cells and bodies in LV midsection and (**d**) in apex of the heart 2 and 4 weeks after LAD-ligation compared to sham treated rats. Scale bars 40 μm. (**e**) Number of white blood cells (WBC) in peripheral blood 2 weeks after LAD-ligation compared to sham. (**f,g**) Cardiomyocyte hypertrophy in LV midsection and (**h**) in apex 2 weeks after LAD-ligation compared to sham. (**i**) Toludine blue staining for mast cells combined with IHC for c-kit. Scale bar 20 μm. N = 5-7 for all groups. Student's t-test was used for comparison between two groups. * $P < 0.05$, ** $P < 0.01$, *** $P < 0.001$.

Supplementary Figure 2. Western blot analysis of expression of c-Kit and SDF1 protein in heart after LAD-ligation. Expression of c-Kit protein (**a**) and SDF1 protein (**b**) in heart 2 weeks after LAD-ligation compared to sham treated rats. GAPDH was used as a loading control. * $P < 0.05$.

Supplementary Figure 1

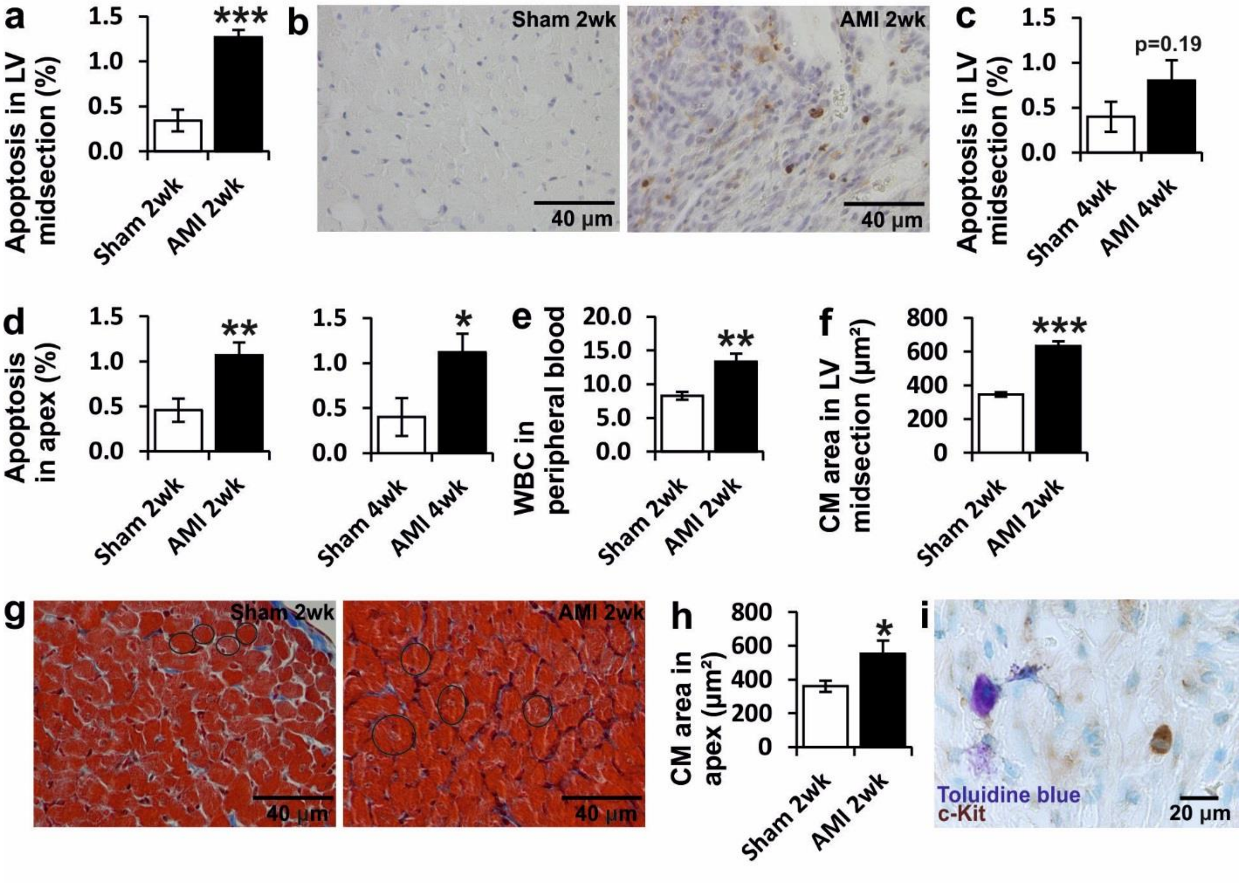

Supplementary Figure 2

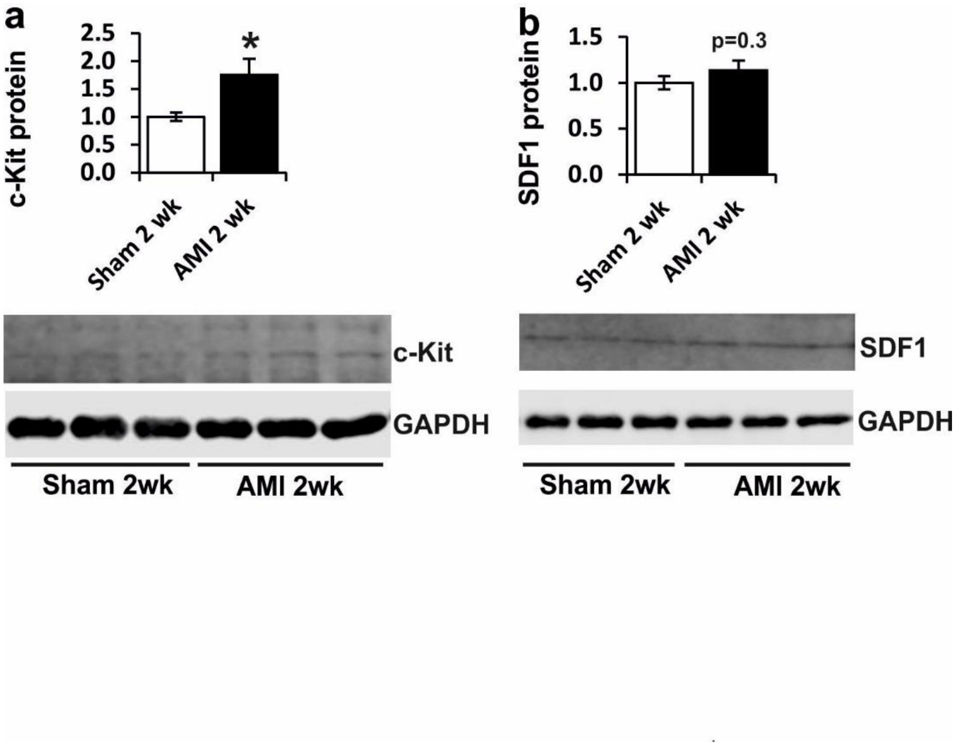

Supplement: Supplementary file 1 — Supplementary information [file 41598_2018_19417_MOESM1_ESM.pdf]
